# Migration, Distribution, and Safety Evaluation of Specific Phenotypic and Functional Mouse Spleen-Derived Invariant Natural Killer T2 Cells after Adoptive Infusion

**DOI:** 10.1155/2021/5170123

**Published:** 2021-12-08

**Authors:** Dongzhi Chen, Wenbin Xu, Jingfang Teng, Huifang Liu, Yuanyuan Wang, Yan Wang, Shujie Cheng, Ming Meng

**Affiliations:** ^1^College of Basic Medicine, Hebei University, Baoding, 071000 Hebei Province, China; ^2^Key Laboratory of Pathogenesis mechanism and control of inflammatory-autoimmune diseases in Hebei Province, Baoding, 071000, Hebei Province, China; ^3^Affiliated Hospital of Hebei University, Baoding, 071000 Hebei Province, China; ^4^Hebei Province Basic Research Key Laboratory of General Surgery for Digital Medicine, Baoding, 071000 Hebei Province, China

## Abstract

Herein, the migration distribution and safety of specific phenotypic and functionally identified spleen-derived invariant natural killer T2 (iNKT2) cells after adoptive infusion in mice were studied. The proliferation and differentiation of iNKT cells were induced by intraperitoneal injection of *α*-galactosylceramide (*α*-GalCer) in vivo. Mouse spleens were isolated in a sterile environment. iNKT cells were isolated by magnetic-activated cell sorting columns (MS columns). Cytometric bead array (CBA) assay was used to detect cytokine secretion in the supernatant stimulated by iNKT cells. The basic life status of the mice was observed, and systematic quantitative scoring was conducted after injecting spleen-derived iNKT cells through the tail vein. An in vivo imaging system was used to trace the migration and distribution of iNKT cells in DBA mice. The percentage of the iNKT2 subgroup was the highest in 3 days after intraperitoneal injection of *α*-GalCer, and iNKT2 subsets accounted for more than 92% after separation and purification by magnetic-activated cell sorting (MACS). Anti-inflammatory cytokine IL-4 was mainly found in the supernatant of cell cultures. The adoptive infusion of iNKT cells into healthy mice resulted in no significant change in the basic life status of mice compared with the noninjected group. iNKT cells were detected in the lung, spleen, and liver, but no fluorescence was detected in lymph nodes and thymus. After dissecting the mice, it was found that there were no significant abnormalities in the relevant immune organs, brain, heart, kidney, lung, and other organs. Intraperitoneal injection of *α*-GalCer results in a large number of iNKT2 cells, mainly secreting anti-inflammatory cytokine IL-4, from the spleen of mice. After adoptive infusion, the iNKT2 cells mainly settled in the liver and spleen of mice with a satisfactory safety profile.

## 1. Introduction

Invariant natural killer T (iNKT) cells are a group of unique immune cells that exhibit the characteristics of both NK and T cells. iNKT cells are restricted by antigen-presenting molecule CD1d, which is of the MHC-I type. *α*-GalCer is a prototype ligand of iNKT cells and a potent stimulator of glycolipid antigens that activates iNKT cells [1–4]. After activation, they quickly secrete a large number of Th1 and Th2 cytokines (IL-2, IL-4, IL-6, IL-10, IL-17, IFN-*γ*, and TNF-*α*) and regulate the differentiation of immune cells and the type of immune response. Because of their cytotoxic effect, they are active in diseases such as cancer and infection and also in type I diabetes, autoimmune encephalomyelitis (EAE), multiple sclerosis (MS), systemic lupus erythematosus (SLE), and other autoimmune diseases, and they play a role in immunosuppression when organs are rejected after transplantation [5–8]. Therefore, adoptive infusion of iNKT cells may be a new strategy for the treatment of cancer, infection, and many immunological diseases.

iNKT cells develop in the thymus, and mature iNKT cells are mainly divided into three subgroups: iNKT1 (secreting IFN-*γ*), iNKT2 (secreting IL-4), and iNKT17 (secreting IL-17) [9–12]. These are widely distributed in the liver, spleen, lymph nodes, lung, fat, and other tissues and organs after maturity. Recent studies have shown that iNKT cell subtypes can more effectively exert its immune regulation and immunotherapy effects when they are specifically activated [13, 14]. iNKT2 cells have shown an increasingly prominent role in the treatment of autoimmune diseases, allergic asthma, and other diseases by releasing anti-inflammatory cytokines, mainly in the form of IL-4. Although iNKT cells play an important role in immune regulation, their small number and low frequency in the body limit their potential in clinical applications [15–18]. Our previous study found that the intraperitoneal injection of *α*-GalCer effectively activates the iNKT2 cell subsets of the spleen. Adoptive infusion of such iNKT2 cells effectively alleviates the clinical symptoms of RA [19, 20]. In the current study, intraperitoneal injection of *α*-GalCer will induce the proliferation of splenic iNKT2 cells, which were subsequently separated and purified in vitro, and test their cytokine secretion function. The isolated and purified iNKT2 cells were adoptively infused into healthy mice. We used an in vivo imaging system of small animals to trace the migration and distribution of iNKT cells in mice and to evaluate the safety of adoptive infusion, so as to provide basic research data for adoptive immunotherapy of iNKT cells.

## 2. Materials and Methods

### 2.1. Experimental Animals

One hundred and sixty 7 to 8-week-old healthy male DBA/1 mice (20.0 ± 1.0 g) were provided by Beijing Vital River Laboratory Animal Technology Co., Ltd. (License No. SCXK (Beijing), 2016-0006). The experiment was carried out after 1 week of adaptive breeding in the SPF animal room. All experiments were approved by the Animal Welfare and Ethical Committee of Hebei University (approval number IACUC-2018017).

### 2.2. Reagents and Instruments

Anti-PE MicroBeads were purchased from Miltenyi (Germany). Th1/Th2/Th17 phenotyping kit, FITC Hamster anti-Mouse TCR- (T cell receptor) *β* chain, PerCP-Cy™5.5 Mouse anti-T-bet (T box expressed in T cells), PerCP-Cy™5.5 Mouse anti-ROR-*γ*t, Alexa Fluor 647 Mouse anti-PLZF (promyelocytic leukemia zinc finger protein), Th1/Th2/Th17 cytokines kit, DiR (1,1-dioctadecyl-3,3,3,3-tetramethylindotricarbocyaine iodide) were purchased from Thermo Fisher Scientific (USA). PE-labeled T-selected-CD1d Tetramer was purchased from MBL International (Japan). KRN7000 (*α*-GalCer) was purchased from AdipoGen. Mouse lymphocyte isolate was purchased from Solarbio (Beijing, China). The MS Columns sorting column was purchased from Miltenyi (Germany). The small animal in vivo imaging system was purchased from PerkinElmer (USA). Phorbol 12-myristate 13-acetate (PMA) and ionomycin (IO) were purchased from Cayman company (USA). Mouse IL-17A, TNF-*α*, IFN-*γ*, and IL-4 cytokine ELISA Kit were purchased from Neobioscience Technology Co, Ltd. (Shenzhen, China).

### 2.3. Isolation and Purification of iNKT Cells by MACS

One-hundred DBA/1 mice were randomly selected from 160 DBA/1 mice, 80 of which were intraperitoneally injected with *α*-GalCer (100 ng/g weight) for 3 days and 20 were intraperitoneally injected with phosphate-buffered saline (PBS). Mouse spleens were harvested under a sterile environment, ground to prepare a single cell suspension, and the lymphocytes were separated using lymphocyte separation medium. After washing twice with PBS, the cells were collected and counted, and each 10^7^ cells were resuspended with 100 *μ*l precooled PBS. Then, 10 *μ*l of PE-labeled CD1d tetramer loaded with *α*-GalCer was added, incubated for 15 min at 4°C in the dark, washed twice with PBS, and resuspended in 80 *μ*l PBS. Next, 20 *μ*l anti-PE microbeads was added, incubated at 4°C for 20 min in the dark, washed twice with PBS, and resuspended in 500 *μ*l PBS. Using magnetic-activated cell sorting (MACS) under sterile conditions, iNKT cells were separated by an MS column.

### 2.4. CBA Detection of Cytokines in Supernatant of Splenic iNKT Cells

Place the purified spleen iNKT cells in a 12-well plate, and resuspend the cells in RPMI-1640 incomplete medium (2 × 10^6^/ml); culture volume was 1.5 ml. Then, PMA (50 ng/ml) and IO (1 *μ*g/ml) were added to the cells. The cells were cultured in the incubator (37°C, 5% CO_2_) for 24 h. The cells were collected and centrifuged to extract the supernatant. The Mouse Th1/Th2/Th17 Cytokine Kit was used to detect the levels of IL-2, IL-17A, TNF-*α*, IL-6, IL-4, IFN-*γ*, and IL-10.

### 2.5. Observation of the Basic Life after Infusion of iNKT Cells from Spleen

Twenty mice were randomly selected from 160 mice and infused with spleen-derived iNKT cells (3 × 10^6^/mouse) via tail vein. Three experimenters observed the vital signs of mice at the same time and independently every day and scored systematically. The average score was used as the final score (Table 1).

### 2.6. Changes of Serum Cytokine Levels in Mice after Infusion of iNKT Cells from the Spleen

Twenty mice were randomly selected and infused with spleen-derived iNKT cells (3 × 10^6^/mouse) via the tail vein. The mouse serum was collected three days later, and the normal mouse serum were used as the control group. The changes of serum cytokines in mice were detected by mouse ELISA kit.

### 2.7. Migration and Distribution of iNKT Cells in DBA Mice Traced by Small Animal In Vivo Imaging System

Twenty mice were randomly selected from 160 mice. DiR (2.5 mg/ml) was dissolved in DMSO and stored at 4°C. Spleen iNKT cells were counted and placed in a 6-well plate. Cells were resuspended in RPMI-1640 incomplete medium (without serum) (1 × 10^6^ cells/ml). DiR (5 *μ*g/ml) solution was added, and the cells were incubated at 37°C under 5% CO_2_ for 25 min. After washing twice with PBS, the cells were resuspended in PBS (5 × 10^6^/300 *μ*l). The DiR-labeled iNKT cells were placed in a black 96-well plate, and the fluorescent labeling of the cells was observed using a small animal in vivo imaging system. Then, the DBA mice were injected with DiR-labeled cells (3 × 10^6^ cells/300 *μ*l/mouse) via the tail vein. At different time points, the small animal in vivo imaging system was used to detect the changes in settlement and migration of iNKT cells after adoptive infusion in mice.

### 2.8. Statistical Analysis

The experimental data were analyzed by SPSS 19.0. (SPSS Inc., Chicago, IL, USA). For all analyses, data were presented as the mean ± SD. In the comparison between the two groups, an unpaired *t*-test was conducted. Differences were considered statistically significant at *P* < 0.05.

## 3. Results

### 3.1. Isolation, Purification, and Identification of iNKT Cells from the Spleen

**Figure 1:**
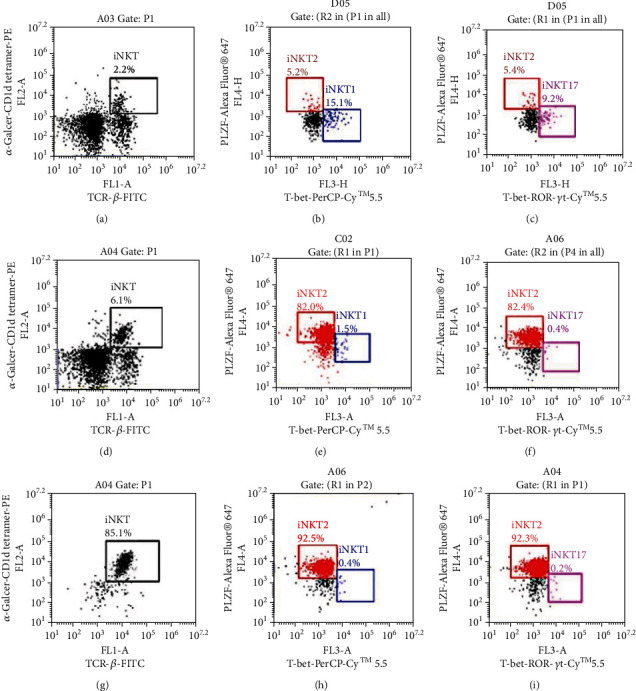
The rates of iNKT cells and proportion of iNKT cell subsets. (a–c) The frequency of iNKT cells and the proportion of iNKT subsets in the spleen of normal mice. (d) The frequency of iNKT cells before purification and three days after injection *α*-GalCer. (e, f) The proportion of iNKT cell subsets before purification and three days after injection of *α*-GalCer. (g) The frequency of iNKT cells after purification and three days after injection of *α*-GalCer. (h, i) The proportion of iNKT cell subsets after purification and three days after injection of *α*-GalCer.

The frequency of iNKT cells in the spleen of normal DBA/1 mice is approximately 2%, of which iNKT2 accounts for 5.2%, iNKT1 accounts for 15.1%, and iNKT17 accounts for 9.2%. The frequency of iNKT cells was approximately 6% after intraperitoneal injection of *α*-GalCer for 3 days, of which the iNKT2 subgroup accounts for 82.0%, iNKT1 accounts for 1.5%, and iNKT17 accounts for 0.4%. The purity of the iNKT cells was greater than 85% after MACS sorting and purification, in which iNKT2 accounted for more than 92%, iNKT1 accounted for 0.4%, and iNKT17 accounted for 0.2% (Figure 1).

### 3.2. Cytokine Secretion in Culture Supernatant of Spleen-Derived iNKT Cells

To understand the function of spleen-derived iNKT cells, we separately isolated normal mouse spleen iNKT cells (control) and mouse spleen iNKT cells (*α*-GalCer) after intraperitoneal injection of *α*-GalCer for 3 days. Detection of cytokine levels in the culture supernatant showed that compared with the normal levels, the levels of inflammatory cytokines (IL-17A, TNF-*α*, IFN-*γ*, IL-6, and IL-2) in the culture supernatant of spleen iNKT cells obtained by intraperitoneal injection of *α*-GalCer were significantly decreased (*P* < 0.05) and the levels of anti-inflammatory cytokines IL-4 were significantly increased (*P* < 0.05). There was no significant difference in IL-10 levels, and the ratio of IFN-*γ*/IL-4 also significantly decreased (*P* < 0.05) (Table 2).

### 3.3. Infusion of iNKT Cells to Observe the Basic Life Status of Healthy DBA/1 Mice

The death rate of healthy DBA/1 mice infused with spleen-derived iNKT cells was zero. The mice were scored based on five points: weight, posture, activity, fur texture, and skin integrity. It was found that after comparison with the control group, there was no significant change in the score of mice as compared with the control group, which showed that adoptive infusion of spleen-derived iNKT cells was safe (Table 3).

### 3.4. Changes of Serum Cytokine Levels in Mice after Infusion of Spleen-Derived iNKT Cells

In order to understand the changes of serum cytokine levels in mice after infusion of spleen-derived iNKT cells, we injected iNKT cells and PBS into the tail vein of normal mice, respectively. Compared with the control group, the serum inflammatory cytokine IL-17A in mice infused with iNKT cells was significantly lower (*P* < 0.05). The levels of TNF-*α* and IFN-*γ* did not change obviously. The level of anti-inflammatory cytokine IL-4 did not change significantly (Table 4).

### 3.5. Migration, Distribution, and Metabolism of iNKT Cells in DBA/1 Mice after Adoptive Infusion

**Figure 2:**
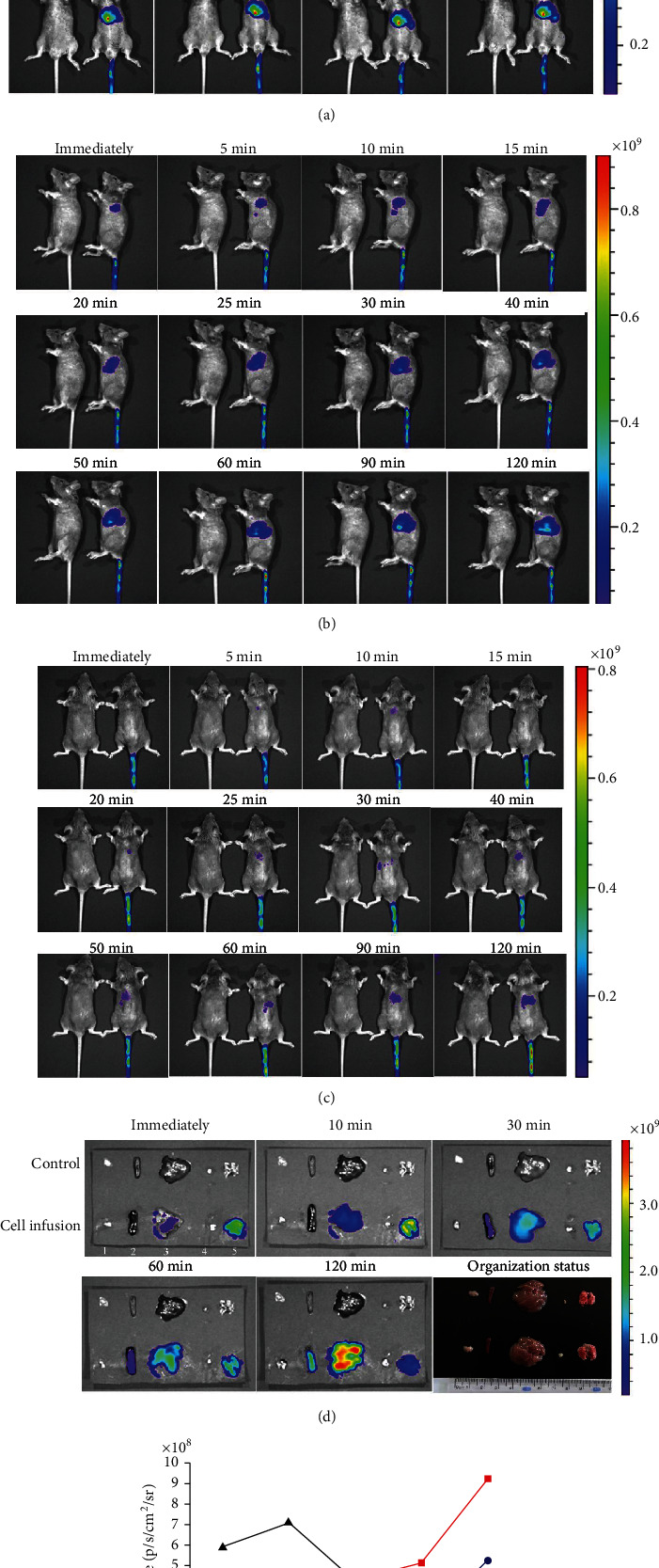
Migration path of iNKT cells tracked by caliper IVIS Lumina II. (a–c) Within 120 min after adoptive infusion of DiR-labeled iNKT cells into mice. The migration path of iNKT cells in mice was monitored from the body surface of mice lying in the supine, lateral, and prone positions. (d) Mice were dissected, and organs were isolated and then detected by fluorescence (iNKT cells infused into the caudal vein first reached the lung, then the liver, and finally the spleen. No fluorescence was detected in the thymus or the inguinal lymph nodes: (1) thymus, (2) spleen, (3) liver, (4) inguinal lymph nodes, and (5) lung). (e) The change in the average fluorescence signal intensity in the spleen, liver, and lung.

Within 120 min after adoptive infusion of DiR-labeled iNKT cells into mice, they were examined to track the appearance of iNKT cells. At immediately 5, 10, 15, 20, 25, 30, 40, 50, 60, 90, and 120 min, the migration path of iNKT cells in mice was monitored from the body surface of mice lying in the supine, lateral, and prone positions. It was found that iNKT cells adoptively injected into mice immediately appeared in the lungs, and fluorescence was detected in the liver at 10 min and in the spleen at 60 min (Figures 2(a)–2(c)).

The mice were dissected at immediately 10,30, 60, and 120 min. The thymus, spleen, liver, groin lymph nodes, and lung were removed for fluorescence intensity detection. It was found that iNKT cells were adoptively infused into mice within 120 min. There was no fluorescence in the thymus or groin lymph nodes. In the lungs, strong fluorescence was detected immediately, and the fluorescence intensity was the strongest at 10 min, but then it gradually weakened. Weak fluorescence was detected in the liver immediately, which then gradually increased. Fluorescence was detected in the spleen at 30 min and then gradually increased (Figures 2(d) and 2(e)).

**Figure 3:**
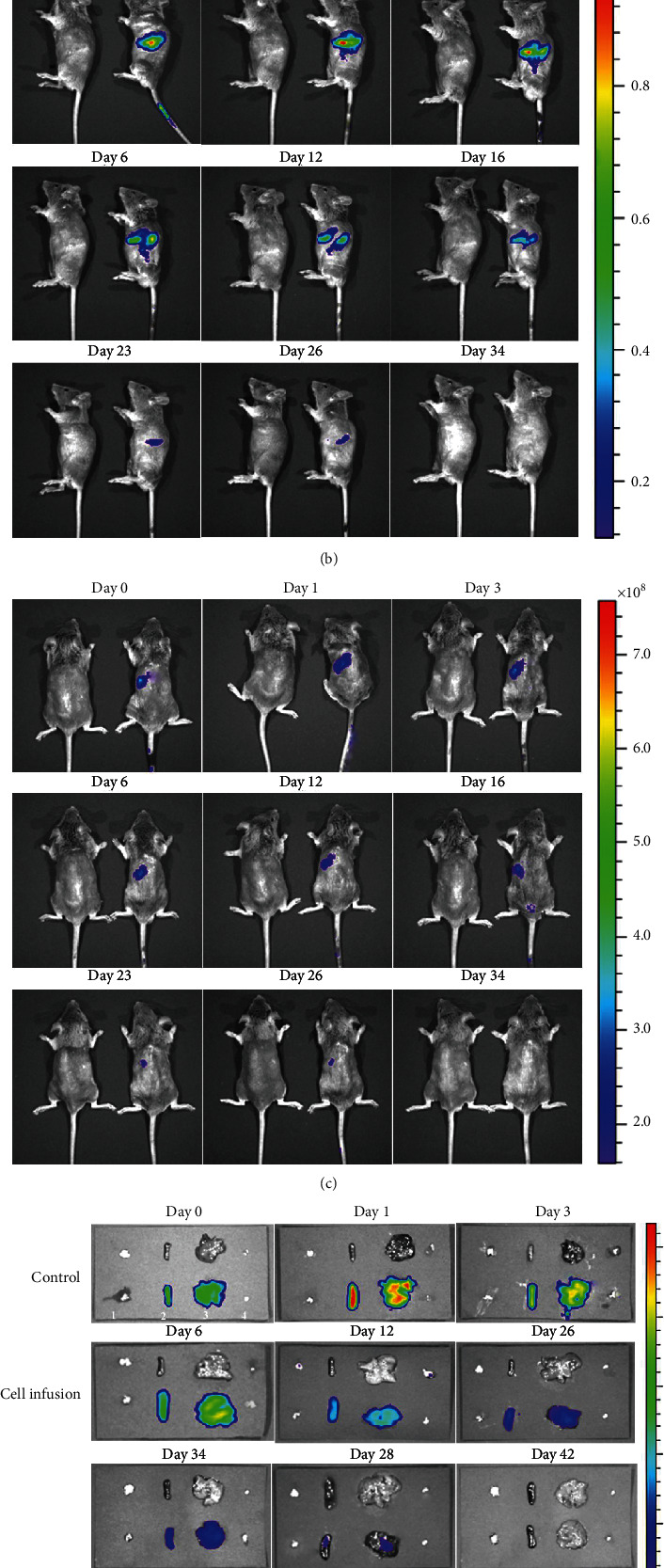
Distribution and metabolism of iNKT cells traced by caliper IVIS Lumina II. (a–c) DiR-labeled spleen-derived iNKT cells were transfused into mice, and the distribution and metabolism (changes in fluorescence intensity) of the iNKT cells were detected. The body surface was monitored from the supine, lateral, and prone positions. (d) Mice were dissected, and organs were isolated and then detected by fluorescence (iNKT cells injected via the tail vein were mainly distributed in the liver and spleen. In vivo detection showed that the fluorescence on body surfaces disappeared at day 34, and in vitro detection showed that the fluorescence of isolated organs disappeared at day 42; (1) thymus, (2) spleen, (3) liver, and (4) groin lymph node). (e) The change in the average fluorescence signal intensity in the spleen and liver.

DiR-labeled spleen-derived iNKT cells were transfused into mice, and the distribution and metabolism (changes of fluorescence intensity) of the iNKT cells was detected at 0 (3 h), 1, 3, 6, 12, 16, 23, 26, and 34 days. From the three positions of supine, lateral, and prone, body surface monitoring indicated that the fluorescence was mainly concentrated in the liver and spleen and disappeared at 34 days after cell infusion (Figures 3(a)–3(c)).

After infusion of DiR-labeled iNKT cells into mice, the mice were dissected at 0 (3 h), 1, 3, 6, 12, 26, 34, 38, and 42 days. The important immune organs (thymus, spleen, liver, and groin lymph nodes) were removed for fluorescence intensity measurement. It was found that there was no fluorescence in the thymus or inguinal lymph nodes after adoptive infusion of iNKT cells into mice, while the spleen and liver exhibited fluorescence. The fluorescence intensity of the spleen and liver was the strongest on the first day after cell infusion, and then, it gradually weakened and disappeared after 42 days. The average fluorescence intensity of the liver was higher than that of the spleen after cell infusion (*P* < 0.05) (Figures 3(d) and 3(e)).

## 4. Discussion

*α*-GalCer is a classic specific activator of iNKT cells extracted from sponge [21, 22], and it can effectively activate iNKT1, iNKT2, and iNKT17. Parekh et al. [23] found that *α*-GalCer exerts a strong activating power that can stimulate all iNKT cells to fully activate and release a large number of Th1-, Th2-, and Th17-type factors together, which results in the so-called “cytokine storm.” After excessive activation, the cells will be in long-term energy. This super-antigen-like property limits the clinical application of *α*-GalCer. Recent studies have shown that the specific activation of iNKT cell subtypes can play a more effective role in immune regulation and immunotherapy [24, 25]. It may be the best choice to select different types of iNKT with specific phenotypes and functions for adoptive therapy according to the pathogenesis of different diseases.

Our previous experiments found that different injection routes of *α*-GalCer can also activate different subtypes of iNKT cells. Subcutaneous injection of *α*-GalCer can induce the proliferation of iNKT1 subsets in the spleen of mice, while intraperitoneal injection of *α*-GalCer can induce massive proliferation of iNKT2 subsets in the spleen of mice. In the current study, normal DBA/1 mice were given *α*-GalCer by intraperitoneal injection to obtain splenic iNKT cells. After sorting by MACS, the frequency of iNKT2 cells exceeded 92%. The culture supernatant contained mainly anti-inflammatory cytokine IL-4. The dynamic balance of proinflammatory and anti-inflammatory cytokines determines the development and outcome of inflammation. Proinflammatory cytokines promote the body's inflammatory damage, while anti-inflammatory cytokines are mainly involved in the body's own defense and tissue repair. Subsequently, we adoptively transfected DiR-labeled spleen-derived iNKT cells with specific phenotypes and functions into normal DBA/1 mice and tracked the migration, distribution, and metabolism of iNKT cells in mice using a small animal imaging technique. Previous studies have reported that tail vein injection results in fluorescence in organs such as the liver and spleen within 30 seconds [26]. We observed that strong fluorescence can be detected in the lungs immediately after cell infusion, and the fluorescence intensity was the strongest at 10 min and then gradually weakened. Weak fluorescence was immediately observed in the liver and gradually increased, but then, the fluorescence intensity gradually decreased after two days, and the fluorescence disappeared after 42 days. Fluorescence was detected in the spleen at 30 min and then gradually increased, and the fluorescence intensity gradually weakened after two days, and the fluorescence disappeared at 42 days.

No fluorescence was detected in the thymus or the inguinal lymph nodes. It is possible that iNKT cells infused through the tail vein did not enter the thymus and lymph nodes or a few iNKT cells may have entered the thymus and lymph nodes, but the number was too small for detection of fluorescence. The fluorescence intensity of the liver and spleen was strong, and the retention time was the longest, which indicated that the infused iNKT cells mainly resided in the liver and spleen. Further comparison showed that the average fluorescence intensity of the liver was always stronger than that of the spleen. This may be due to the vigorous metabolism and rich blood flow of the liver, which is more suitable for the residence of iNKT cells [27]. The infusion of iNKT cells into the liver may be the most effective and may adequately provide reference values for adoptive therapy of iNKT cells in liver-related diseases. No fluorescence was found in adipose tissue. We will explore the distribution of iNKT in adipose tissue in future studies.

The mortality rate of mice after adoptive infusion of cells was zero. The mice were scored based on five points: weight, posture, activity, fur texture, and skin integrity. It was found that there was no significant change in the scores of mice when compared to the group that did not receive cell infusion. No abnormalities were found in relevant immune organs or the brain, heart, kidney, lung, or other organs after dissection of mice. After infusion of iNKT cells into healthy mice, there was no significant change in serum cytokines except IL-17A, and there was no increase in inflammatory response in mice, indicating that adoptive infusion of spleen-derived iNKT2 cells was safe.

## 5. Conclusions

Intraperitoneal injection of *α*-GalCer results in a large number of iNKT2 cells that mainly secrete anti-inflammatory cytokine IL-4 from the spleen of mice. After adoptive infusion, the iNKT2 cells mainly settled in the liver and spleen of mice, with a satisfactory safety profile.

## Figures and Tables

**Table 1:** Standard for clinical score of mice with iNKT infusion.

Criteria	Grade 0	Grade 1	Grade 2
Weight loss	<10%	>10% to <25%	>25%
Posture	Normal	Only lift the spine while stretching the body	The spine has been bulging heavily
Activity	Normal	Mild to moderate reduction	Only stimulation can activate
Fur texture	Normal	Mild to moderate wrinkles	Severely wrinkled or sparse
Skin integrity	Normal	Scales on claws or tail	Obvious skin peeling

**Table 2:** Cytokine levels in the culture supernatant of mouse spleen-derived iNKT cells (pg/ml).

Cytokines	Control	*α*-GalCer
Proinflammatory cytokine		
IL-17A	15.37 ± 0.16	2.62 ± 0.47^∗^
TNF-*α*	33.42 ± 0.49	0.77 ± 0.22^∗^
IFN-*γ*	15.57 ± 0.27	1.87 ± 0.03^∗^
IL-6	41.21 ± 0.27	11.94 ± 0.41^∗^
IL-2	1.34 ± 0.10	1.39 ± 0.15
Anti-inflammatory cytokine		
IL-4	61.18 ± 1.02	110.09 ± 0.55^∗^
IL-10	20.08 ± 0.24	20.50 ± 0.23
Ratio		
IFN-*γ*/IL-4	0.24 ± 0.02	0.02 ± 0.01^∗^

^∗^ *P* < 0.05 vs. control.

**Table 3:** Clinical score of mice with iNKT infusion.

Day	0 (3 h)	1	3	6	12	16	23	26	34	38	42
Control	0	0	0	0	0	0	0	0	0	0	0
Cell infusion	0	1	0.7	0.3	0	0	0	0.3	0	0	0

**Table 4:** Serum cytokine levels in healthy mice infused with iNKT cells (pg/ml).

Cytokines	Control	Cell infusion
Proinflammatory cytokine		
IL-17A	112.70 ± 5.48	66.94 ± 10.14^∗^
TNF-*α*	132.83 ± 13.32	141.17 ± 6.03
IFN-*γ*	20.46 ± 3.69	25.61 ± 11.67
Anti-inflammatory cytokine		
IL-4	25.05 ± 1.58	30.31 ± 7.70

^∗^ *P* < 0.05 vs. control.

## Data Availability

The data used to support the findings of this study are available from the corresponding author upon request.
